# Gene Expression Profiling and Immune Pathway Dysregulation in Ribonucleoprotein Autoantibody-Positive Systemic Lupus Erythematosus Patients

**DOI:** 10.3390/genes15111353

**Published:** 2024-10-22

**Authors:** Siyuan Tan, Tirupapuliyur Damodaran

**Affiliations:** 1 School of Life Sciences, Shandong University, Jinan 250012, China; 202200141039@mail.sdu.edu.cn; 2 Department of Biological and Biomedical Sciences, North Carolina Central University, Durham, NC 27707, USA

**Keywords:** systemic lupus erythematosus, RNP autoantibodies, gene expression, immune regulation

## Abstract

Background: Systemic lupus erythematosus (SLE) is an autoimmune disorder characterized by immune dysregulation and chronic inflammation across various organ systems. While anti-dsDNA and anti-Sm antibodies are commonly associated with SLE, the presence of anti-RNP antibodies is often linked to unique gene expression profiles and immune responses. This study aims to investigate the gene expression profiles in ribonucleoprotein (RNP) autoantibody-positive SLE patients by analyzing publicly available transcriptomic data. Methods: This study analyzed transcriptomic data from the GEO dataset GSE61635, which includes gene expression profiles from 79 anti-RNP-positive SLE patients and 30 healthy controls. Differentially expressed genes (DEGs) were identified using the GEO2R tool with a *p*-value < 0.05 and |log2fold change| > 1. Gene Ontology (GO) and Kyoto Encyclopedia of Genes and Genomes (KEGG) enrichment analyses were performed. Tissue-specific and cell-type enrichment analyses highlighted the involvement of immune tissues. Results: A total of 1891 DEGs were identified between anti-RNP-positive SLE patients and healthy controls. Among the identified DEGs, *SLC4A1* and *EPB42* were notably downregulated, while *PIP4K2A* was highly upregulated. Enrichment analyses revealed significant dysregulation in antiviral response and immune regulation pathways. PPI network analysis highlighted key hub genes, suggesting a heightened antiviral state in these patients. Tissue-specific enrichment and cell-type enrichment identified the bone marrow and immune tissues as being highly affected by the altered gene expression. Additionally, gene frequency analysis highlighted *RASD2* as being recurrently significant across multiple studies. Conclusions: The findings suggest that anti-RNP-positive SLE patients exhibit distinct gene expression and immune dysregulation profiles, particularly in antiviral and immune regulation pathways. These results provide insights into the molecular mechanisms driving SLE in this patient subset and may guide future therapeutic interventions.

## 1. Introduction

Systemic lupus erythematosus (SLE) is a complex, chronic autoimmune disease characterized by a breakdown in self-tolerance, resulting in immune responses against multiple organ systems, including the skin, kidneys, and joints [1]. Despite advances in understanding the immune dysfunctions of SLE, many questions remain regarding the specific gene expression patterns that link immune dysregulation to clinical manifestations.

The presence of various autoantibodies is an important hallmark in SLE. Selected antinuclear antibodies (ANAs), including anti-double-stranded deoxyribonucleic acid (dsDNA) and anti-Sm, are highly specific for the diagnosis of SLE. Anti-RNP antibodies are present in a subset of patients with SLE, and high-titer anti-RNP antibodies are present in all patients with mixed connective tissue disease, a disorder that is closely related to SLE [2]. Although anti-RNP antibodies are not a primary diagnostic criterion for SLE, their association with mixed connective tissue diseases and their potential link to specific immune dysregulation warrant investigation. This study aims to explore the gene expression profiles in anti-RNP-positive SLE patients to identify the pathways that might differentiate them from other serotypes, potentially revealing novel insights into SLE pathogenesis.

Previous research has primarily focused on the role of the adaptive immune system, particularly T cells and B cells, in SLE pathogenesis. B cells play a critical role in the disease by not only producing autoantibodies, but also acting as antigen-presenting cells [3]. T cells, especially CD4+ helper T cells, are key drivers of immune responses, facilitating the activation of B cells and the production of pathogenic autoantibodies, including anti-nuclear antibodies (ANAs) [4]. These autoantibodies contribute to immune complex formation, which is a hallmark of SLE and a primary cause of tissue inflammation and damage to ANAs [5]. The interaction between these two immune cell types leads to chronic inflammation and subsequent organ damage.

More recently, attention has shifted towards the role of the innate immune system, especially type I interferons (IFNs) and plasmacytoid dendritic cells (pDCs), in SLE pathogenesis. The persistent production of type I IFNs, particularly IFN-α, is a major feature of SLE and has been shown to play a critical role in disease progression. This is largely driven by immune complexes containing nucleic acids, which activate pDCs to produce IFN-α, contributing to a loss of tolerance and the activation of autoreactive T and B cells [6,7].

Genetic predisposition also plays a significant role in SLE, with several studies identifying associations between susceptibility to SLE and polymorphisms in genes involved in the interferon pathway. In recent years, high-throughput technologies such as transcriptomic and microarray analyses have been widely applied to identify differentially expressed genes (DEGs) in SLE [8]. These approaches have facilitated the identification of potential biomarkers and therapeutic targets by providing insight into the molecular pathways involved in disease progression. Numerous studies have highlighted the dysregulation of genes involved in immune responses, including those associated with interferon signaling, as critical contributors to SLE pathogenesis.

Despite advances in understanding the immune dysfunctions of SLE, many questions remain regarding the specific gene expression patterns that link immune dysregulation to clinical manifestations. In this study, we seek to explore these gaps by analyzing gene expression in RNP autoantibody-positive SLE patients using publicly available datasets. Using bioinformatic tools, we screened DEGs from GSE61635 and performed Gene Ontology (GO) and Kyoto Encyclopedia of Genes and Genomes (KEGG) pathway enrichment analyses to identify the key biological processes and pathways involved in SLE. A PPI network was constructed to identify hub genes, and further tissue- and cell type-specific enrichment analyses were performed to investigate the potential roles of these genes in disease progression. The insights gained from this study provide a deeper understanding of the molecular underpinnings of SLE and may guide future therapeutic strategies.

## 2. Materials and Methods

### 2.1. Data Source

The GEO dataset GSE61635, which profiles gene expression in RNP autoantibody-positive SLE patients versus healthy controls, was used for this study. The dataset includes mRNA expression data from 79 SLE patients and 30 healthy volunteers.

RNA samples were collected from all patients following the provision of informed consent. The cohort consisted of 73 female and 6 male subjects. The duration of disease among the patients ranged from 0 to 453 months, with a median duration of 37.5 months. The SLE Disease Activity Index (SLEDAI) scores varied between 0 and 31, with a median score of 6 [9].

### 2.2. Data Processing

To identify differentially expressed genes (DEGs) within the GSE61635 dataset, we employed the GEO2R tool, a user-friendly web-based application that allows for the comparison of two or more groups of samples within the GEO series. GEO2R uses the Limma (Linear Models for Microarray Data) package to identify DEGs by comparing expression levels across different conditions. The selection criteria for DEGs were set as a *p*-value less than 0.05 and an absolute log2 fold change greater than 1. The DEGs identified through this process were considered for further downstream analyses.

### 2.3. Functional Enrichment Analysis

To understand the biological significance of the identified DEGs, we conducted KEGG (Kyoto Encyclopedia of Genes and Genomes) and GO (Gene Ontology) enrichment analyses. We utilized the KOBAS (KEGG Orthology-Based Annotation System) tool (Version 3.0, Peking University, Beijing, China, available at http://bioinfo.org/kobas/; accessed on 3 August 2024). By inputting the DEG list into KOBAS, we identified pathways and gene functions that were significantly enriched, providing insight into the underlying biological mechanisms.

### 2.4. PPI Network Building

To further understand the functional interactions between the identified DEGs, we constructed a protein–protein interaction (PPI) network. This was accomplished using STRING (Search Tool for the Retrieval of Interacting Genes/Proteins) and Cytoscape, (Version 3.10.2) a robust bioinformatics software platform for visualizing molecular interaction networks. Initially, DEGs were input into the STRING database to predict interactions based on evidence from experimental data, co-expression, and other sources. The interaction data from STRING were then imported into Cytoscape to build and visualize the PPI network.

### 2.5. Tissue Enrichment Analysis

Tissue enrichment analysis was conducted to determine the specific tissues where DEGs are most highly expressed. We used the TissueEnrich R package for this purpose [10]. This package employs a statistical framework to analyze gene expression data, focusing on identifying the enrichment of genes within specific tissues. The input for this analysis is DEGs identified in the earlier transcriptome analysis step. The TissueEnrich package uses the GTEx dataset as a reference for tissue-specific gene expression profiles, allowing for the identification of significant tissue associations of DEGs.

### 2.6. Gene Frequency Analysis

Gene frequency analysis was performed to identify the recurrence of specific genes across different studies using the same GEO dataset (GSE61635) [11,12,13,14,15,16,17,18]. This helps in highlighting genes that are consistently reported as significant, suggesting their potential biological importance. Key genes were extracted from related studies that had employed GSE61635. For each gene, the frequency of its occurrence was calculated across these studies, which provided a quantitative measure of its relevance and consistency.

### 2.7. Cell Type Annotation

We used Enrichr (https://maayanlab.cloud/Enrichr/; accessed on 23 August 2024) for the cell type annotation on the upregulated and downregulated genes, and identified the primary cell types associated with each group. These results provided further insights into the cellular mechanisms involved in SLE progression.

## 3. Results

### 3.1. Differential Gene Expression Analysis

**Figure 1:**
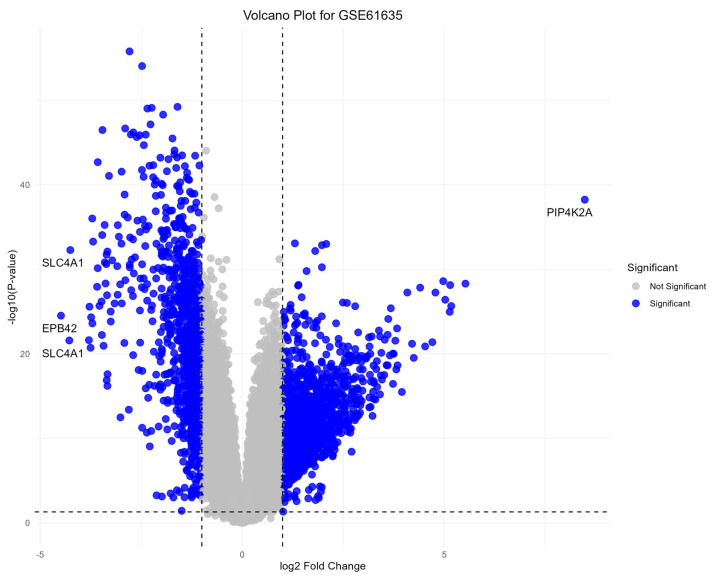
Volcano plot of DEGs between SLE and control groups.

A total of 1891 DEGs (*p*-value < 0.05, |log2fold change| > 1) were identified between RNP autoantibody+ SLE patients and healthy blood donors. The volcano plot illustrates the distribution of upregulated and downregulated genes (Figure 1). Notably, *SLC4A1* and *EPB42* are strongly downregulated with a log2fold change of less than −4, while *PIP4K2A* is highly upregulated with a log2fold change greater than 8. These genes represent the most significant expression changes in the dataset, suggesting their potential involvement in disease mechanisms.

### 3.2. Functional Enrichment Analysis Results

**Figure 2:**
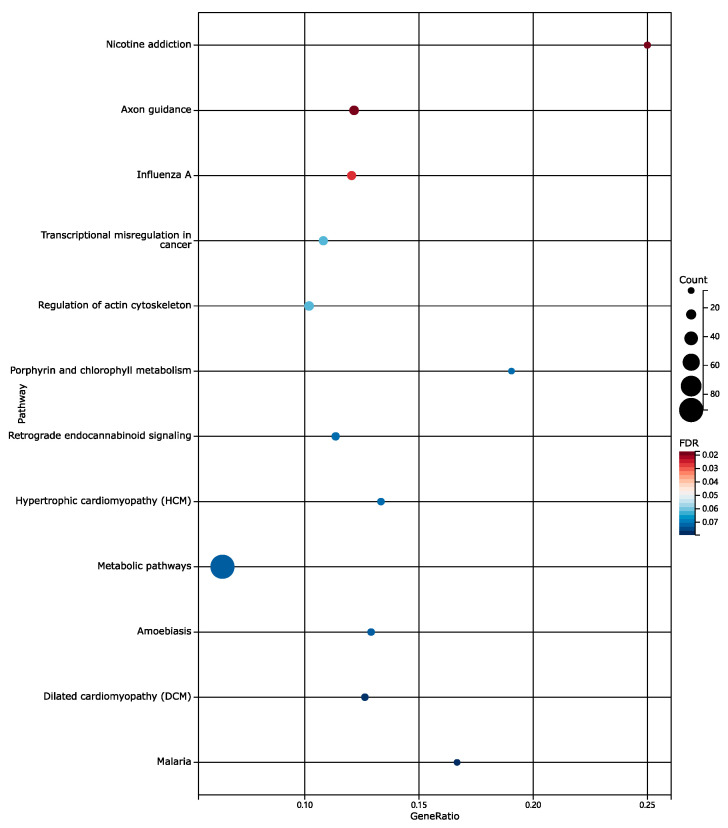
KEGG pathway enrichment analysis of DEGs in RNP autoantibody+ SLE patients. The size of each bubble corresponds to the number of genes enriched in each pathway, while the color gradient represents the significance level (*p*-value) of the enrichment, with darker colors indicating higher statistical significance.

**Figure 3:**
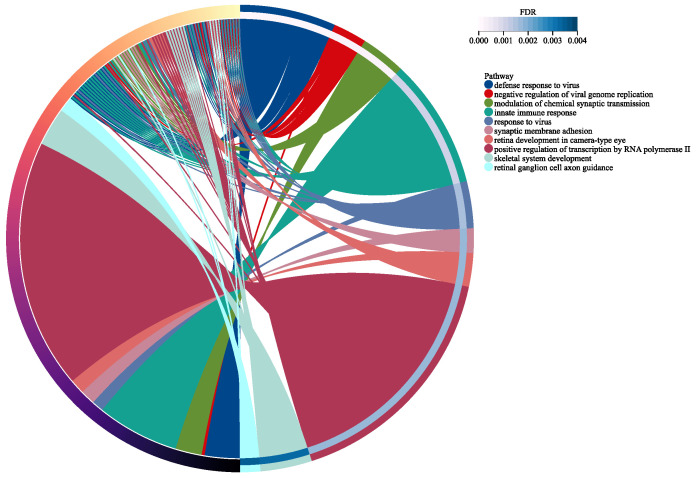
GO enrichment analysis of DEGs in RNP autoantibody+ SLE patients. The chord diagram represents the GO enrichment analysis results, displaying the relationships between enriched GO terms and their associated DEGs.

To further understand the biological processes and pathways associated with the DEGs, we performed KEGG enrichment analysis (Figure 2) and GO enrichment analysis (Figure 3) using the KOBAS tool.

Multiple pathways related to antiviral response were highly enriched, suggesting a heightened immune response against viral infections in RNP autoantibody+ SLE patients. This is consistent with previous studies indicating a link between viral infections and SLE pathogenesis. Pathways involved in immune regulation, such as innate immune response and the modulation of chemical synaptic transmission, were also significantly enriched. This indicates the dysregulation of immune responses in RNP autoantibody+ SLE patients, contributing to the development and progression of the disease. Pathways related to nicotine addiction and influenza A were also enriched. This suggests potential interactions between environmental factors and gene expression in SLE patients.

### 3.3. Protein–Protein Interaction (PPI) Network Analysis

**Figure 4:**
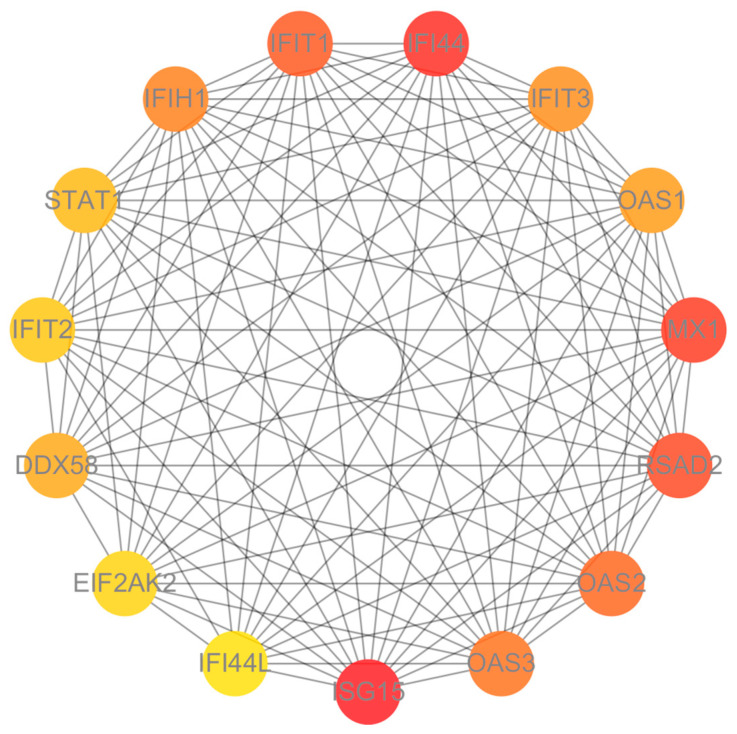
Protein–protein interaction (PPI) network of top 15 hub genes. The color of the nodes reflects the degree of connectivity, and edges indicate protein–protein interactions, with darker nodes representing higher connectivity.

We constructed a PPI network using STRING and Cytoscape to identify hub genes and explore their functional relationships (Figure 4). The network includes 15 hub genes. The overexpression of these hub genes in the SLE group suggests a heightened antiviral state and altered immune regulation in RNP autoantibody+ SLE patients.

### 3.4. Tissue Enrichment Analysis Results

**Figure 5:**
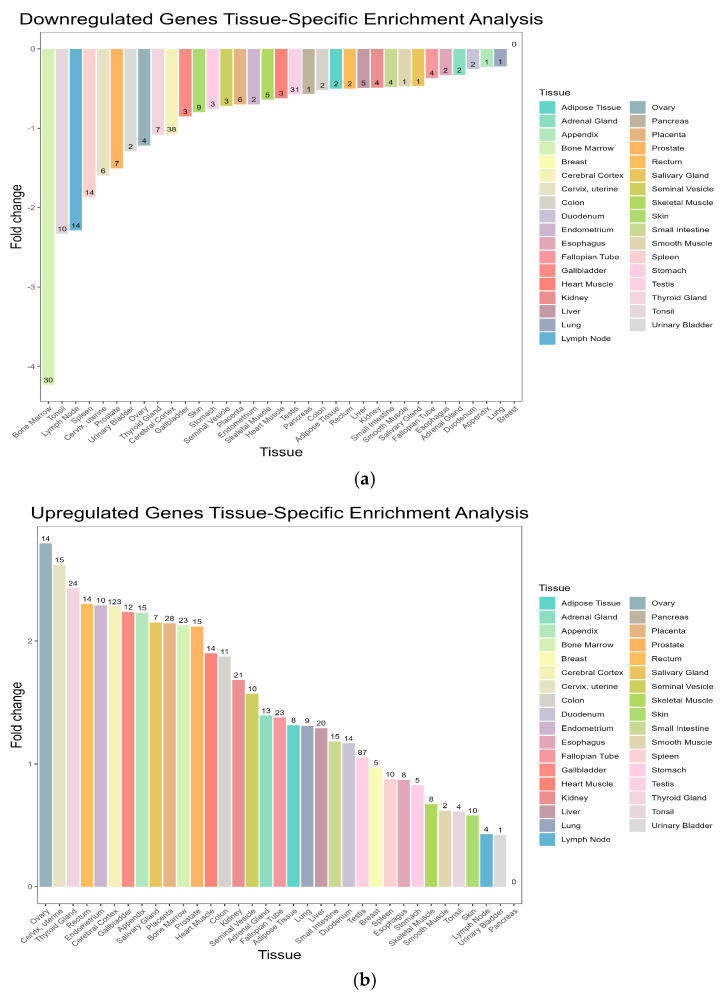
(**a**) Tissue-specific enrichment analysis of downregulated genes in RNP autoantibody+ SLE patients. (**b**) Tissue-specific enrichment analysis of upregulated genes in RNP autoantibody+ SLE patients. The numbers displayed above each tissue bar represent the number of genes enriched in each tissue.

We performed tissue enrichment analysis to explore the tissue-specific expression patterns of the DEGs (Figure 5). The results revealed a significant enrichment of DEGs in immune tissues, such as bone marrow, as well as reproductive tissues, such as prostate and endometrium. This suggests that SLE may have a broader impact on various organ systems beyond the immune system.

### 3.5. Gene Frequency Analysis Results

**Figure 6:**
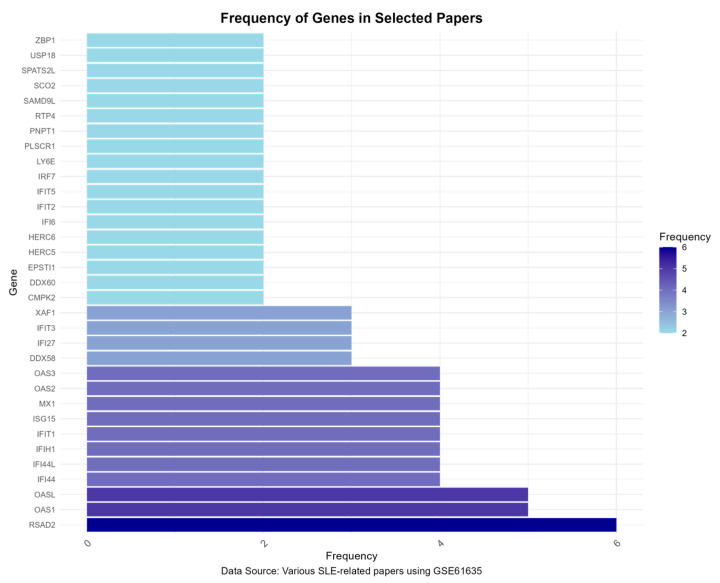
Gene frequency analysis of key genes identified in multiple studies using the GSE61635 dataset.

To validate the findings and prioritize genes for further investigation, we conducted a gene frequency analysis using data from selected studies utilizing the same dataset (GSE61635). This analysis revealed a high frequency of several key genes across different studies, with *RASD2* standing out in particular, further supporting its critical role in SLE pathogenesis (Figure 6).

## 4. Discussion

This study explores gene expression changes in systemic lupus erythematosus, providing important insights into both immune dysfunction and red blood cell (RBC) function. By analyzing differentially expressed genes, we identified several key pathways and mechanisms that may play a role in systemic lupus erythematosus progression, with immune cell dysfunction and RBC instability emerging as central contributors.

Tissue-specific enrichment analysis revealed significant gene expression changes in the bone marrow, cerebral cortex, and testes, suggesting these tissues also play crucial roles in disease pathogenesis. The bone marrow is a primary site for immune cell production, and disruptions here may reflect altered lymphocyte development. Changes in the cerebral cortex may contribute to the neurological manifestations seen in SLE, while alterations in the testes highlight potential reproductive health impacts in male patients [19,20].

One notable finding is the downregulation of genes critical for RBC stability, including *SLC4A1* and *EPB42*. *SLC4A1* is responsible for maintaining membrane integrity and facilitating CO_2_ transport, while *EPB42* supports the shape and mechanical properties of red blood cells [21,22]. The decreased expression of these genes may explain symptoms like anemia and fatigue, expanding the conventional view of systemic lupus erythematosus as not just an immune disorder, but also one that affects RBCs.

Our tissue and cell type enrichment analyses revealed distinct patterns of immune cell regulation. Downregulated genes were found mainly in granulocytes and myeloid cells, suggesting that innate immunity may be compromised, contributing to chronic inflammation and increased susceptibility to infections in patients with systemic lupus erythematosus [23,24]. On the other hand, upregulated genes were predominantly associated with inflammatory cells, such as M1 macrophages and neutrophils, which is consistent with the heightened inflammatory response typically seen in the disease [25]. These findings align with previous research that suggests an overactivation of the innate immune system contributes to tissue damage in systemic lupus erythematosus [26].

Previous studies have shown that B cells not only produce pathogenic autoantibodies, but also act as antigen-presenting cells, further activating T cells and perpetuating the autoimmune response. T cells, especially the CD4+ and CD8+ subsets, have been implicated in driving direct tissue damage and contributing to the inflammatory response [7]. While much of the previous research has focused on the adaptive immune response—particularly involving T and B cells—our results highlight the importance of innate immunity and RBC dysfunction in the disease. This broader understanding opens new possibilities for diagnosis and treatment, suggesting that addressing both immune dysregulation and RBC instability could help alleviate symptoms like anemia.

However, this study has its limitations. First, we relied on publicly available GEO datasets, which may affect the generalizability of our findings due to variations in populations and study conditions. Second, the accuracy of tissue enrichment analyses depends on the quality of reference datasets, which could introduce bias. Most importantly, we did not perform functional experiments to validate the roles of the identified genes. Future research will need to conduct such experiments to confirm how these genes contribute to systemic lupus erythematosus and their impact on both immune cells and RBCs.

In the future, functional studies such as gene knockdown or overexpression experiments should be conducted to determine the precise role of these genes in systemic lupus erythematosus. Additionally, larger studies involving diverse patient populations and more detailed immune cell analyses are necessary to fully understand the complexity of the disease and to identify new therapeutic targets.

## 5. Conclusions

In this study, we explored the distinct gene expression profiles and immune dysregulation in anti-RNP-positive SLE patients, identifying significant alterations in immune and antiviral response pathways. The analysis of DEGs, including the downregulation of SLC4A1 and EPB42 and the upregulation of PIP4K2A, highlighted potential molecular mechanisms contributing to SLE pathogenesis, particularly in antiviral pathways. Our findings also underscore the involvement of tissues such as bone marrow and immune cells in the disease process, further suggesting that both immune and red blood cell dysfunction play pivotal roles in the progression of SLE. These insights open new avenues for therapeutic intervention, potentially targeting these dysregulated pathways to alleviate both immune and hematological symptoms in patients. Future studies should focus on functional validation of these key genes and their roles in SLE progression to enhance our understanding of disease mechanisms and treatment strategies.

## Data Availability

The authors declare that the data supporting the findings of this study are available within the paper.
